# Recent Developments of Acoustic Energy Harvesting: A Review

**DOI:** 10.3390/mi10010048

**Published:** 2019-01-11

**Authors:** Ming Yuan, Ziping Cao, Jun Luo, Xiujian Chou

**Affiliations:** 1 School of Automation, Nanjing University of Posts and Telecommunications, Nanjing 210023, China; 2 School of Telecommunications and Information Engineering, Nanjing University of Posts and Telecommunications, Nanjing 210023, China; luojun@njupt.edu.cn; 3 Science and Technology on Electronic Test and Measurement Laboratory, North University of China, Taiyuan 030051, China

**Keywords:** acoustic energy harvesting, energy conversion, wave manipulation, smart materials and structures

## Abstract

Acoustic energy is a type of environmental energy source that can be scavenged and converted into electrical energy for small-scale power applications. In general, incident sound power density is low and structural design for acoustic energy harvesting (AEH) is crucial. This review article summarizes the mechanisms of AEH, which include the Helmholtz resonator approach, the quarter-wavelength resonator approach, and the acoustic metamaterial approach. The details of recently proposed AEH devices and mechanisms are carefully reviewed and compared. Because acoustic metamaterials have the advantages of compactness, effectiveness, and flexibility, it is suggested that the emerging metamaterial-based AEH technique is highly suitable for further development. It is demonstrated that the AEH technique will become an essential part of the environmental energy-harvesting research field. As a multidisciplinary research topic, the major challenge is to integrate AEH devices into engineering structures and make composite structures smarter to achieve large-scale AEH.

## 1. Introduction

Acoustic sound waves are mechanical waves that possess energy and can be generated by many noise sources. When the sound wave is undesired, we refer to it as noise. Common noise sources include airplanes, vehicles, high-speed trains, power plants, loudspeakers, machines, and expressways.

Nowadays, we live in a noisy world, and acoustic energy is an indispensable environmental energy source. The environmental sound levels can be higher than 100 dB, and low-frequency noise is dominant in the frequency spectrum [1,2,3,4,5].

Generally, acoustic energy is ultimately dissipated into thermal energy at the propagation stage, and low- and midfrequency sound waves have attracted the most attention. One reason is that this frequency band of noise is usually a significant component of the spectrum. The other reason is that in this frequency range, the corresponding sound wavelength is long, making it difficult to absorb or isolate it using most engineering structures. Many approaches have been developed to effectively absorb or isolate low- to midfrequency acoustic sound waves, and these include passive approaches [6,7,8,9,10,11,12] and active approaches [13,14,15,16,17]. 

At present, the target is to convert sound energy into electrical energy rather than dissipate it. Compared to other environmental energy types (solar, wind, and hydroelectric), acoustic energy is less influenced by the weather and can be harvested with fewer installation restrictions. The drawback of acoustic energy is that its power is low. This means that, when acoustic energy is converted into electrical energy, the power level cannot meet the demand of high power-consuming applications. On the other hand, with the rapid development of modern electrical technologies, the power requirements of embedded chips are substantially decreasing, so that the harvested power can be supplied to low-power devices [18]. 

Acoustic waves belong to one kind of mechanical waves and utilizing ambient energy sources has always been a hot topic in recent years. For instance, an up-to-date review article comprehensively summarized vibration-based energy-harvesting approaches from various of energy sources [19]. In this well-organized review, vibration sources are classified into different categories. Furthermore, it emphasized that resonance is an essential element to realize energy harvesting from continues vibrations or acoustic waves. With respect to the acoustic energy-harvesting (AEH) technique, because environmental acoustic waves belong to weak excitation sources, the AEH device’s design is different from that of the vibration-based energy harvester. Acoustic resonance is crucial in AEH design, which can be used to amplify the incident sound wave and sufficiently excite energy conversion part.

With the help of AEH technology, distributed smart sensing and condition monitoring applications are anticipated for innovative applications. In addition, the harvested acoustic energy can provide a trigger function and be utilized as an alarm.

To successfully convert acoustic energy into electrical energy, an AEH apparatus is required. The incident acoustic energy can be focused by such a device and piezoelectric, electromagnetic, or triboelectric effects can be used for energy conversion. After conversion, a power-managing circuit is used for voltage rectification [20], regulation, and impedance matching. At the peripheral side, a power-saving unit, such as a supercapacitor, is used for energy storage. 

**Figure 1:**
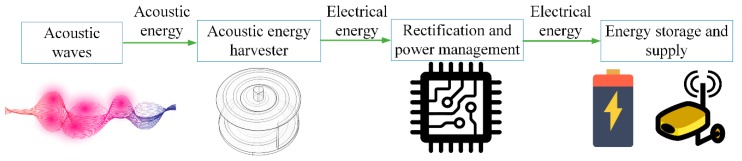
Schematic diagram of an acoustic energy-harvesting (AEH) system.

The schematic diagram of an AEH system is shown in Figure 1.

In this review, recently established AEH approaches are reviewed, which aims to pave the way for their further development. To provide a better understanding of this technology, we first present the basic elements of audible acoustics. Subsequently, the AEH mechanism is described because it is an essential part of AEH device design. In Section 4, a detailed review of AEH studies is presented, and the performances of the AEH devices are summarized in Section 5. At the end of the article, we provide comments on current issues and perspectives concerning AEH research. 

## 2. Audible Acoustic Fundamentals

### 2.1. Sound Propagation in Air

Here, the frequency range of interest is 20 Hz–20 kHz, which is audible to humans. In the air, sound is propagated in a longitudinal wave format, and the mathematical equation for plane wave sound propagation is:

(1) $$\frac{\partial^{2}p}{\partial x^{2}}-\frac{1}{c^{2}}\frac{\partial^{2}p}{\partial t^{2}}=0$$

Propagation speed c in the air is 343 m/s (20 °C) and the relationship between sound speed, wavelength, and frequency is expressed as:

*c* = *λ*·*f*, (2)

where f is the frequency and λ is the wavelength.

In general, we treat sound propagation as adiabatic and inviscid. However, when sound is propagated in narrow tubes or small-scale places, the viscous loss considerably contributes to acoustic damping. 

The thickness of viscous layer $\delta_{v}$ has a close relationship with the frequency, which is expressed as [21]:

(3) $$\delta_{v}=\sqrt{\frac{2\mu }{\rho_{0}\omega }}$$

where μ is the dynamic viscosity, $\rho_{0}$ is the air density, and ω is the angular frequency. For 100 Hz sound in air, the thickness of the viscous layer can be up to 0.22 mm. Therefore, to harvest acoustic energy in low frequencies, decreasing the influence of viscous layer is favorable.

### 2.2. Sound Pressure Level and Sound Power

Audible sound pressure greatly varies and the metric logarithmic-scale sound-pressure level (SPL) is usually used as an effective measurement index. The detailed expression of the SPL is given as [22]:

(4) $$\mathrm{SPL}=20\log_{10}(p/p_{ref})$$

where p is the measured root-mean-squared sound pressure, and $p_{ref}$ is the reference sound pressure, which equals 20 µPa in the air. The SPL’s unit is decibel.

Sound pressure p can be linked to sound intensity I (acoustic power per unit area in the perpendicular direction) in a further step. For the plane wave propagation case, the relationship is given in Equation (5):

(5) $$I=p\cdot v=p^{2}/Z$$

where the particle velocity is v and the specific acoustic impedance is Z.

Then, sound power Pow (to avoid confusion with sound pressure p), which is the product of sound intensity I and incident surface area A, can be represented as:

(6) $$Pow=I\cdot A=(p^{2}\cdot A)/Z$$

Table 1 list typical values of sound pressures and the corresponding SPL and sound intensity values.

According to the sound-power definition, sound power is proportional to the squared sound pressure value. Furthermore, because of the logarithmic scale, it has been shown that sound intensity can significantly vary even if SPL differences are small.

## 3. Acoustic Energy Harvesting (AEH) Mechanism

### 3.1. Acoustic-Wave Manipulation

In general, environmental sound pressure is low. For instance, when the SPL value equals 100 dB, the corresponding sound pressure equals only to 2 Pa. If no measures are taken, such weak excitation cannot sufficiently excite small-scale structures.

Therefore, in AEH studies, an acoustic resonator is usually used to augment sound pressure to achieve strong excitation of the structure. 

**Figure 2:**
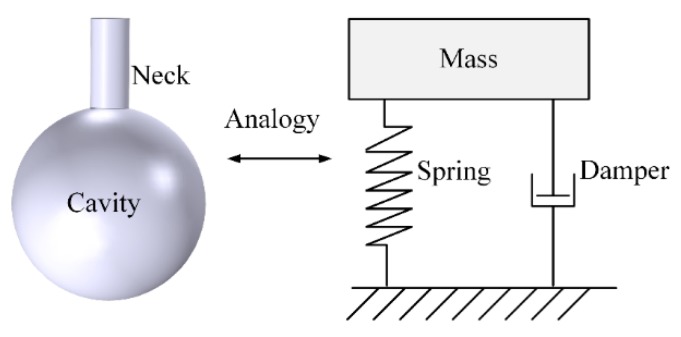
Schematic diagram of a Helmholtz resonator and its mechanical analogy.

For instance, the classical Helmholtz acoustic resonator provides an effective method to amplify incident sound waves. The Helmholtz resonator consists of a neck and a cavity, and the resonator walls are assumed to be rigid. The acoustic system is equivalent to a single degree of freedom (SDOF) mechanical system for the lower mode. A schematic diagram of a Helmholtz resonator is shown in Figure 2.

The SDOF system indicates that this device can achieve strong sound-pressure amplification at a specific frequency, which indicates the occurrence of acoustic resonance. According to the lumped element method, its fundamental acoustic resonant frequency can be calculated as:

(7) $$f=\frac{c}{2\pi }\sqrt{\frac{s}{V(l+\gamma a)}}$$

where c is the sound speed, s is the neck’s cross-sectional area, l is the neck length, V is the cavity volume, *a* is the radius of the open neck, and γ is a correction factor, which is assumed to be 0.82 [23].

**Figure 3:**
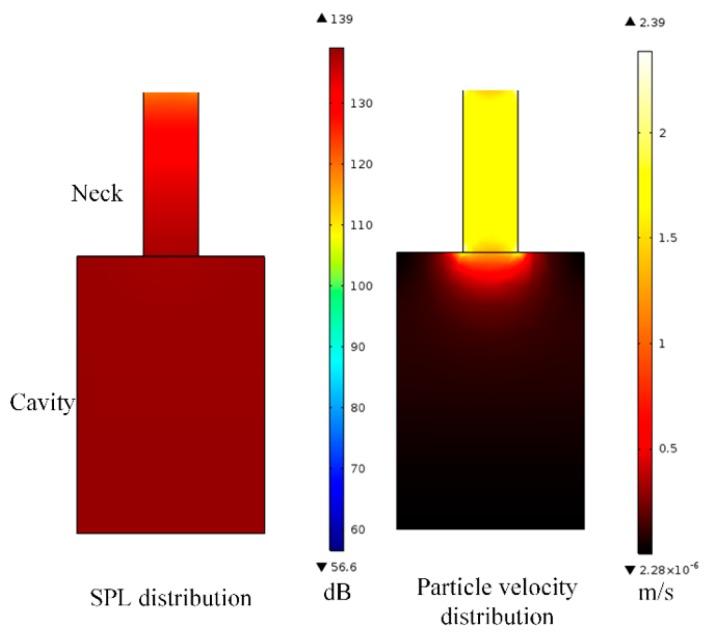
Sound pressure and velocity distribution of a Helmholtz resonator at the resonant frequency.

A finite-element simulation of a Helmholtz resonator was developed using COMSOL^TM^ to demonstrate the SPL and velocity distribution, as shown in Figure 3.

At the acoustic resonance state, sound pressure increases gradually from the inlet to the cavity, and sound-pressure distribution inside the cavity is nearly uniform. To achieve AEH, the bottom of the Helmholtz resonator is usually flexible. This generates sufficient pressure difference to the ambient sound pressure, which is favorable in the AEH design. In addition, particle velocity is substantially higher at the neck (Figure 3), and this contributes to major viscous loss, which was stated in Section 2.1. The viscous damping causes the resonator’s measured sound-pressure amplification ratio to be smaller than the theoretical value. 

Another commonly used acoustic resonator, which is called the quarter-wavelength tube resonator, has also been used to amplify incident sound pressure. When the tube’s length is equal to the quarter wavelength of the incident sound wave, the reactance part of the acoustic system becomes zero and acoustic resonance will occur. 

**Figure 4:**
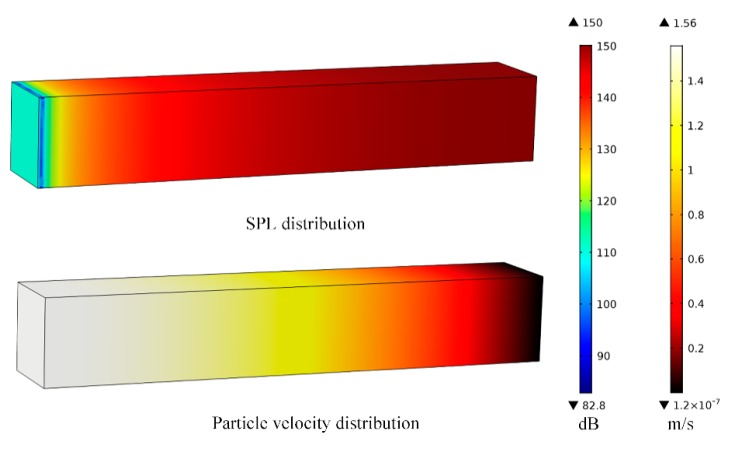
Sound-pressure level (SPL) distribution and particle-velocity distribution of a 0.6 m long quarter-wavelength tube resonator at 143 Hz.

For instance, for a 0.6 m long quarter-wavelength tube resonator, at resonance, the corresponding acoustic wavelength equals to 2.4 m, which corresponds to 143 Hz resonant frequency. SPL distribution and particle-velocity distribution inside the tube under this condition are shown in Figure 4. 

These figures provide important information on acoustic resonators, so that the energy-conversion parts can be appropriately located to achieve effective AEH design.

Aside from the aforementioned classical approaches, rapidly developing acoustic metamaterials, which are artificial structures, exhibit extraordinary abilities for sound-wave manipulation. Acoustic metamaterials are powerful devices for sound-wave manipulation and their properties, which include sound-pressure amplification, local resonance, subwavelength scaling, and energy focusing, are favorable for developing AEH devices. Several relevant review papers have been published on acoustic metamaterials and provide readers with a quick and clear overview of this topic [24,25,26,27,28]. Specifically, energy harvesting using metamaterials is elaborated on in References [19,28] and provides a solid foundation for this field.

### 3.2. Energy Conversion

When acoustic waves are impinging on the AEH device, acoustic energy is usually converted into elastic-strain energy through a fluid–structure interaction process [29,30,31]. Subsequently, an energy converter is essential to transform this kind of energy into electrical energy. For AEH applications, piezoelectric and electromagnetic materials are the most common approaches described in the AEH literature.

#### 3.2.1. Piezoelectric Conversion

Piezoelectric material can transform the mechanical strain energy into electrical energy through the piezoelectric effect. Commonly used piezoelectric materials, which include polyvinylidene difluoride (PVDF), lead zirconate titanate (PZT), and barium titanate (BaTiO3), are summarized in Table 2 [32]. 

Piezoelectric coefficient $d_{31}$ significantly influences energy-conversion efficiency because the achieved electromechanical coupling factor is proportional to piezoelectric coefficient $d_{31}$.

In addition, the single-crystal piezoelectric material has attracted the attention of researchers [33]. The single crystal piezoelectric material achieves much higher electromechanical coupling than the commonly used PZT-5A and PZT-5H materials, thereby increasing the energy harvesting performance significantly. 

The highly oriented PZT film also exhibits a strong piezoelectric effect [34,35], which generates sufficient strain under low-frequency conditions and weak excitations. These materials could be applied to AEH, but more investigations are required. 

#### 3.2.2. Electromagnetic Conversion

Following Faraday’s law of electromagnetic induction, when a permanent magnet is moved relative to a coil, an electrical potential is created at the coil’s terminals. Induced voltage V is proportional to the change in the rate of magnetic flux density B and the turns of coil n:

(8) V=−n⋅(dB/dt)

Because the magnetic field is created by a permanent magnet, the magnetic field has to have a high value of remanence permeability $B_{r}$. Nowadays, the most commonly used type of magnetic material is neodymium iron boron (NdFeB), which is the strongest magnetic material available for commercial use. NdFeB is usually graded using numbers and a larger grade number indicates a higher $B_{r}$ value. For instance, for the N30 grade, the $B_{r}$ value is 1.08∼1.15 T and, for the N52 grade, the $B_{r}$ value is 1.42∼1.47 T.

## 4. Established AEH Approaches

Many studies have investigated AEH mechanisms. In this section, based on the type of acoustic-wave manipulation, the mechanisms are classified into Helmholtz resonator-based approaches, quarter-wavelength resonator-based approaches, and acoustic metamaterial based-approaches. A review of existing studies using these mechanisms is presented.

### 4.1. Helmholtz Resonator-Based Approaches

**Figure 5:**
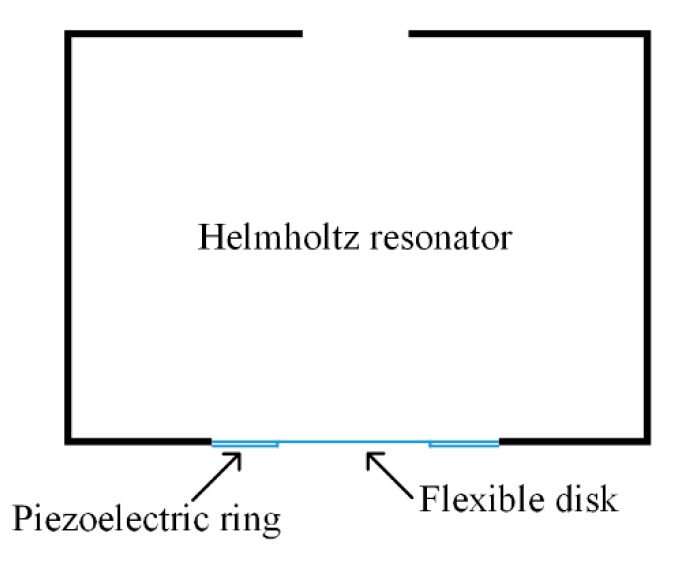
Schematic diagram of a Helmholtz resonator with a compliant bottom; a piezoelectric ring serves as the converter. This represents a MEMS-scale device.

This intuitive concept is based on the pioneering AEH research by Horowitz et al. [36]. As shown in Figure 5, a coupled resonant system is established to achieve AEH at the MEMS scale. Although the reported energy-harvesting power is low, this approach demonstrates the feasibility of AEH.

**Figure 6:**
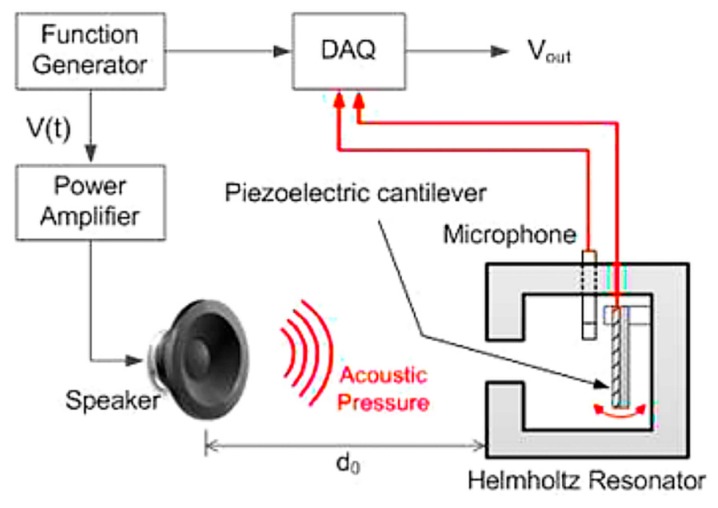
Polyvinylidene difluoride (PVDF) cantilever beam inside the Helmholtz resonator to achieve AEH. Reproduced with permission from [37]; published by Springer, 2013.

Noh et al. [37] fabricated a PVDF cantilever beam, which was placed inside the cavity of the Helmholtz resonator to achieve AEH. The schematic diagram of the proposed device is shown in Figure 6. Using this approach, 0.1 µW power was harvested. The very small amount of harvested energy was attributed to two reasons, i.e., the low piezoelectric coefficient of the PVDF material and the acoustic resonant frequency; internal sound-pressure distribution in the Helmholtz resonator was uniform (Figure 3), indicating that the net excitation strength of the cantilever beam was weak. 

**Figure 7:**
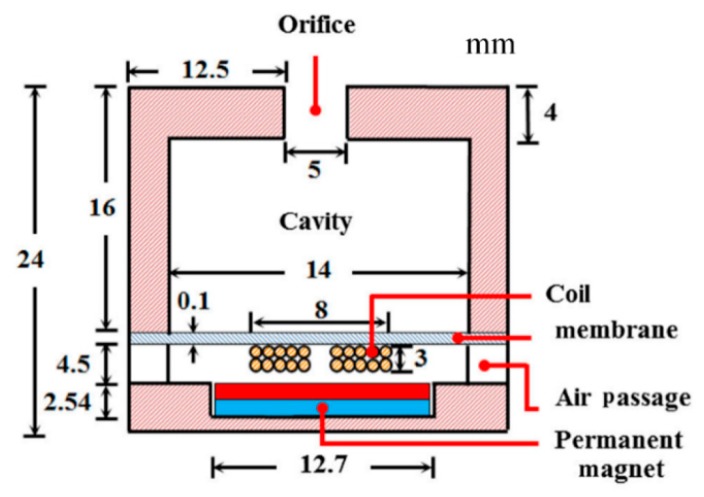
Schematic diagram of the electromagnetism-based AEH device; a coil is placed at the resonator’s bottom and a permanent magnet is placed inside a holder. Reproduced with permission from [38]; published by Springer, 2016.

A permanent magnet can be placed at the bottom of the Helmholtz resonator, resulting in an electromagnetic AEH device. As shown in Figure 7, a coil is placed on the flexible bottom and a magnet is placed in a holder; the coil and magnet are separated by an air passage [38].

To improve the Helmholtz resonator-based AEH performance, several improved approaches were subsequently developed. 

**Figure 8:**
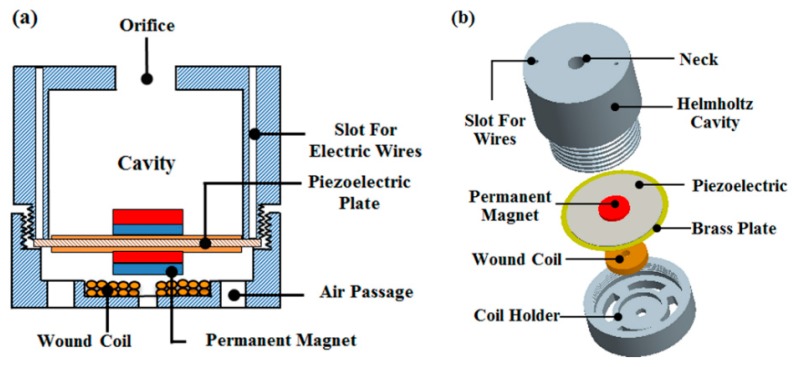
Schematic diagram of the hybrid AEH device, which is based on the Helmholtz resonator and integrates piezoelectric and electromagnetic conversion. (**a**) Cross-section view; (**b**) exploded view. Reproduced with permission from [39]; published by American Institute of Physics, 2016.

A hybrid AEH device, which integrates the piezoelectric and electromagnetic conversion approaches, was proposed by Khan and Izhar [39]. As shown in Figure 8, the air passage in the AEH device was specifically designed to minimize the air damping influence. The harvested power was 49 µW (from the piezoelectric part) and 3.16 µW (from the electromagnetic part) at 2100 Hz, and excitation strength was 130 dB.

**Figure 9:**
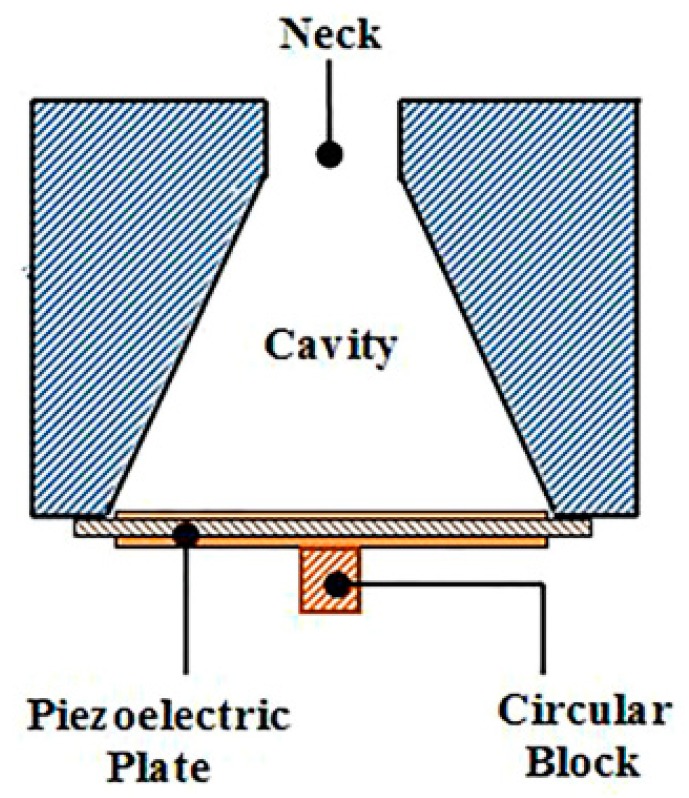
Schematic diagram of the proposed AEH, which is equipped with a conical Helmholtz resonator and a piezoelectric conversion device. Reproduced with permission from [40]; published by American Institute of Physics, 2016.

Khan and Izhar [40] also proposed a conical Helmholtz resonator for AEH. Unlike the classical Helmholtz resonator, the conical configuration ensures that a higher sound-pressure amplification ratio can be achieved. As the pressure difference of the flexible bottom increases, AEH performance is improved. The schematic diagram of the proposed device is shown in Figure 9.

**Figure 10:**
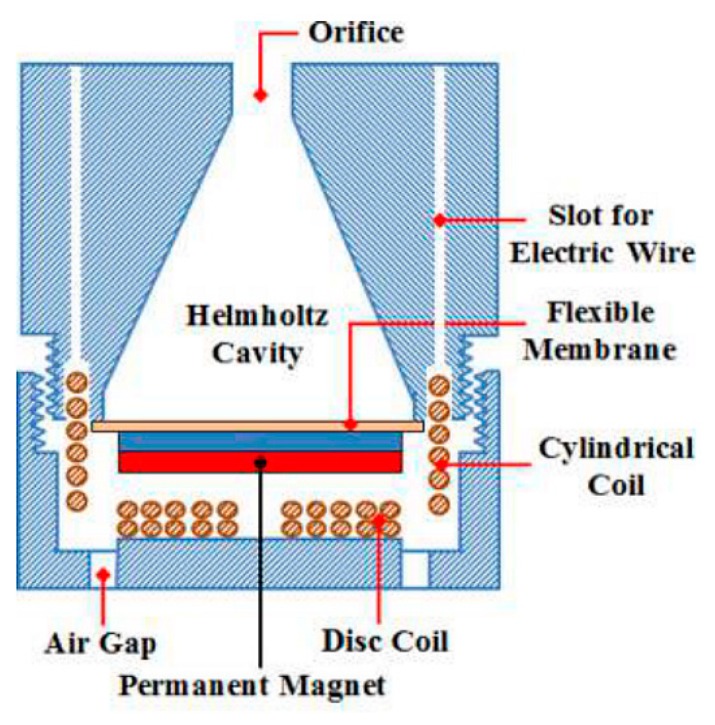
Schematic diagram of the proposed AEH, which is equipped with a conical Helmholtz resonator and an electromagnetic conversion device. Reproduced with permission from [41]; published by Emerald Insight, 2018.

A conical Helmholtz resonator with an electromagnetic conversion device was proposed by Reference [41]; the schematic diagram of the device is shown in Figure 10. 

AEH performance can also be improved by resonant coupling. For instance, a dual Helmholtz structure, consisting of neck–cavity–neck–cavity components, was proposed to enhance the acoustoelectric coupling [42]. A modified version of the device was used to extend the energy-harvesting bandwidth [43].

**Figure 11:**
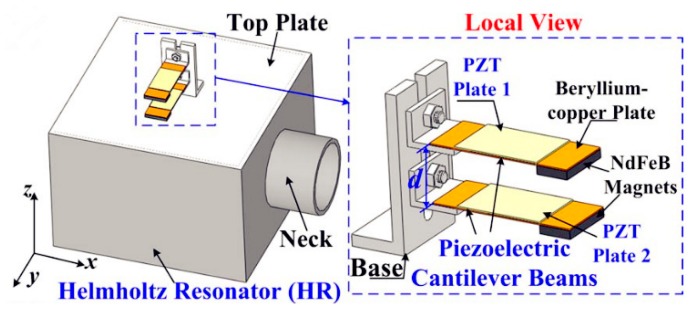
Schematic diagram of the AEH system with strong coupling. Reproduced with permission from [44]; published by American Institute of Physics, 2014.

If the resonant frequency of the mechanical portion is tuned to the same frequency as the acoustical resonant frequency, the harvesting efficiency can be improved substantially. For instance, Yang et al. [44] utilized two cantilever beams and a Helmholtz resonator to achieve strong acoustical–mechanical coupling. The system’s schematic diagram is shown in Figure 11; experiment results demonstrated that a maximum of 1.43 mW power could be extracted for 100 dB SPL excitation at 170–206 Hz. The cantilever beam can also be combined with a Helmholtz resonator with a tapered cavity, which is favorable to extend the bandwidth of the AEH system [45].

**Figure 12:**
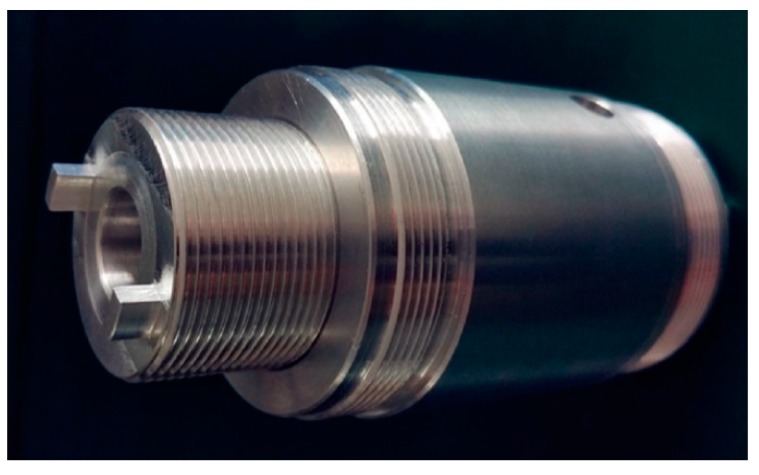
Photograph of the tunable neck to increase AEH efficiency. Reproduced with permission from [46]; published by ELSEVIER, 2017.

A tunable neck is an alternative approach to achieve resonant coupling. As shown in Figure 12, neck length is variable, which changes the acoustic resonance frequency. Specifically, the acoustic resonance can be tuned to match the bottom’s mechanical resonance, thereby increasing the AEH performance substantially [46].

**Figure 13:**
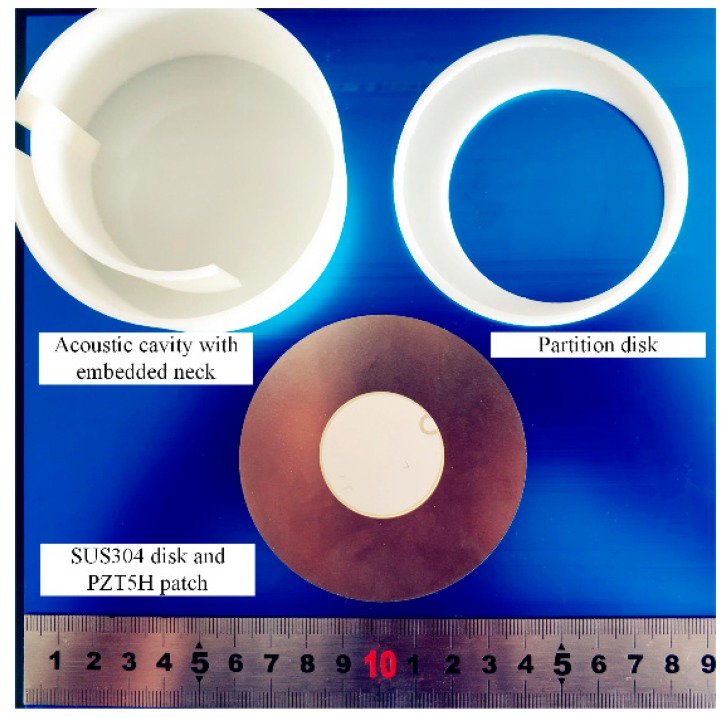
Photograph of the components of the tapered AEH device [47].

The traditional Helmholtz resonator is bulky for low-frequency applications. To overcome this drawback, Yuan et al. [47] proposed a planar Helmholtz AEH device for a low-frequency range. As shown in Figure 13, an embedded coiled neck is used to make the resonator compact. In addition, a tapered neck is used to reduce the viscous loss along the neck. The results demonstrated that this configuration was favorable to improve the resonator’s performance and generated 27.2 µW power at 217 Hz and 100 dB SPL excitation.

**Figure 14:**
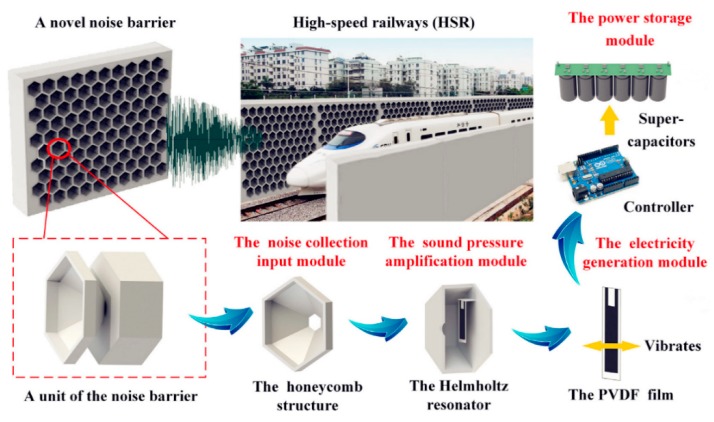
Components of the AEH unit and the array form to create a noise barrier. Reproduced with permission from [48]; published by ELSEVIER, 2018.

A multifunctional noise barrier, which simultaneously achieves sound insulation and AEH, was designed, fabricated, and evaluated by Wang et al. [48]. As shown in Figure 14, a PVDF film was used for energy conversion in this application because the PVDF material has a low piezoelectric coefficient and, in a single unit, the harvested power was only 0.38 µW under 100 dB of excitation. If multiple units were used, a noise barrier could be created and the harvested power would substantially increase. 

**Figure 15:**
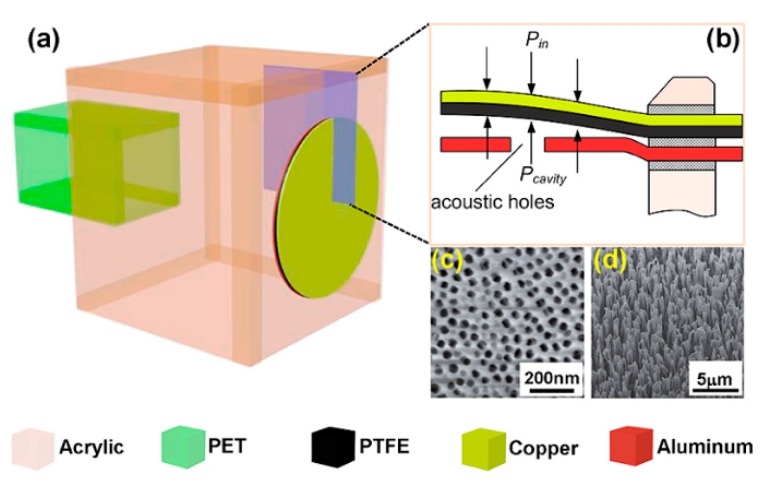
Schematic diagram of the nanogenerator-based AEH device. (**a**) AEH Structure; (**b**) cross-sectional view of the triboelectric nanogenerator (TENG); (**c**) SEM image of the pores on the electrode; (**d**) SEM image of the nanowires. Reproduced with permission from [49]; published by ACS NANO, 2014.

Yang et al. [49] proposed a triboelectric nanogenerator (TENG) for AEH. As shown in Figure 15, the nanogenerator was placed on a Helmholtz resonator, which can be excited strongly at the resonant frequency. The tribolectric charges result in increases in voltage. 

Subsequently, Cui et al. [50] demonstrated an improved version of the TENG with good wideband AEH performance and durability. 

### 4.2. Quarter-Wavelength Resonator-Based Approaches

**Figure 16:**
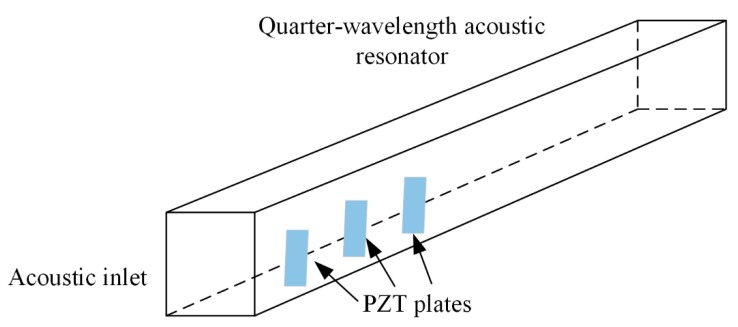
Schematic diagram of quarter-wavelength resonator tube and the installed PZT beams.

Li et al. [51] placed multiple PVDF beams in a quarter-wavelength tube resonator and achieved low-frequency AEH. It was found that the beam placed at the inlet generated the maximum power and the zigzag beam placement resulted in better performance than the aligned configuration. Later, Li et al. [52] used PZT beams to replace the PVDF beams, and experimental tests showed a significant improvement in the AEH performance. The schematic diagram of the quarter-wavelength tube resonator and installed PZT beams is shown in Figure 16. 

Subsequently, Li and You [53] designed a self-powered synchronized switch harvesting on inductor (SSHI) circuit and integrated it into a quarter-wavelength resonator-based AEH device. It was demonstrated that the self-powered S-SSHI circuit achieved the best performance and the harvested power was 0.796 mW under 112 dB SPL excitation at 196 Hz.

### 4.3. Acoustic Metamaterial Based-Approaches

Acoustic metamaterials provide many valuable approaches to develop AEH structures. One of the important applications of acoustic metamaterials is to obtain sound pressure focusing or amplification. This mechanism has inspired several AEH studies.

Wu et al. [54] used sonic crystals with a point defect to confine acoustic energy at the center; a PVDF patch was placed at the defect to convert acoustic energy into electrical energy. 

**Figure 17:**
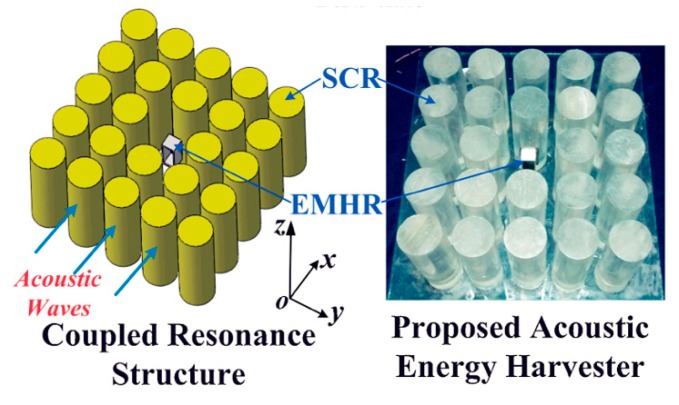
Coupled AEH system consisting of a sonic crystal resonator (SCR) and electromechanical Helmholtz resonator (EMHR). Reproduced with permission from [55]; published by the Japan Society of Applied Physics, 2016.

Later, Yang et al. [55] designed an improved performance prototype based on the sonic crystal resonator (SCR). As shown in Figure 17, a delicate electromechanical Helmholtz resonator (EMHR) was then placed at the center, which resulted in strong acoustical–mechanical coupling. The coupled system achieved 23 and 262 times higher performance than the EMHR and SCR structures, respectively.

**Figure 18:**
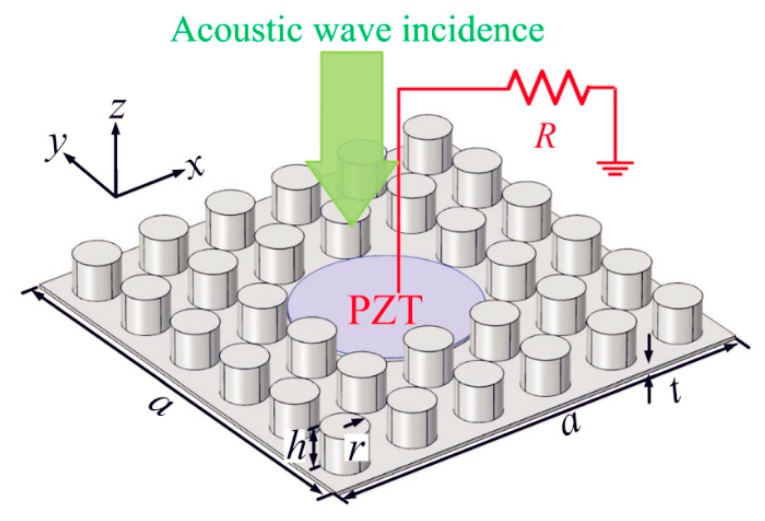
Schematic diagram of the planar acoustic metamaterial-based AEH. Reproduced with permission from [56]; published by American Institute of Physics, 2016.

To reduce harvesting frequency, Qi et al. [56] proposed a planar acoustic metamaterial-based approach for AEH, which is shown in Figure 18. The large strain energy is converted into electrical energy using a bonded piezo patch. The proposed structure also resulted in satisfactory sound-insulation performance.

Later, a subwavelength defect was created by Oudich and Li [57] using an acoustic metamaterial plate to confine the plate’s elastic energy to the defect. The simulation results showed that, at a size of 3 × 3 cm^2^, 18 µW electrical power was harvested under 520 Hz and 100 dB acoustic excitation.

**Figure 19:**
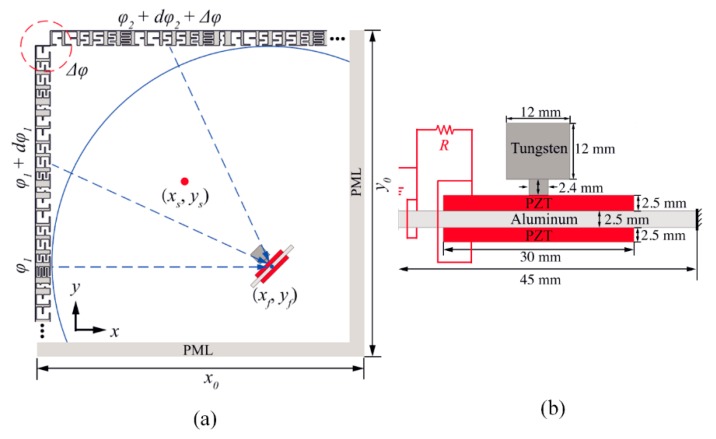
(**a**) Schematic diagram of the metasurface; the piezo beam is placed at the sound-focusing location. (**b**) Detailed piezo beam description. Reproduced with permission from [58]; published by American Institute of Physics, 2017.

Qi and Assouar [58] utilized an acoustic multilateral metasurface to achieve sound focusing. As shown in Figure 19, a piezoelectric bimorph was placed at the confined sound energy point, resulting in strong excitation and AEH.

**Figure 20:**
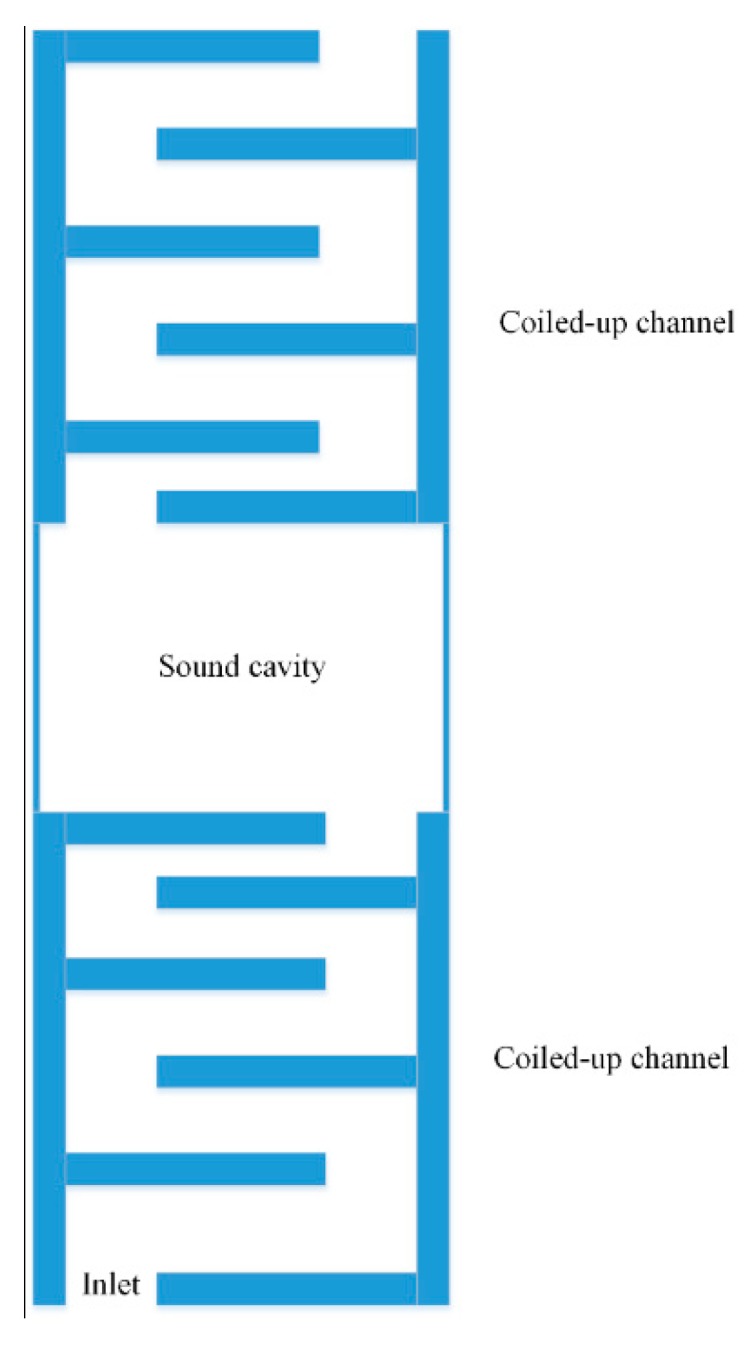
Configuration of the proposed sound energy-harvesting system.

Sun et al. [59] proposed a double coiled-up acoustic cavity for sound pressure enhancement. Inside the cavity, a piezoelectric bimorph plate is used for AEH. The schematic diagram of the device is shown in Figure 20.

**Figure 21:**
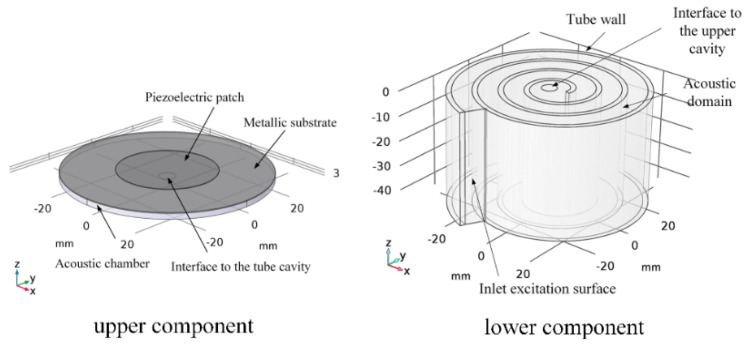
Schematic diagram of the helix structure AEH prototype. Reproduced with permission from [60]; published by American Institute of Physics, 2018.

Another advantage of acoustic metamaterials is that they produce a deep subwavelength effect in acoustic devices, which is favorable to develop small-scale AEH devices for low frequencies. For instance, Yuan et al. [60] proposed a helix-type AEH structure that represents one type of bionic acoustic metamaterials [61]. The helix structure changes the sound propagation path and achieves low-frequency AEH in a compact design. The components of the helix-type AEH structure are shown in Figure 21. This device can harvest 7.3 µW of electrical power at 175 Hz under 100 dB SPL excitation. 

Acoustic metamaterials also exhibit local resonance characteristics. Accordingly, thin structures can exhibit extraordinary sound-insulation performance in the low-frequency range. In addition, when local resonance occurs, substantial strain energy is generated from the metacell, thereby enabling AEH.

For instance, Ma et al. [62] developed an acoustic metasurface with a hybrid resonance that not only resulted in perfect absorption but also AEH. A permanent magnet was used to efficiently convert acoustic energy into electrical energy.

Later, Li et al. [63] proposed a membrane acoustic metamaterial for AEH. The experimental results showed a close relationship between energy-conversion efficiency and tensile stress. However, because the application of precise tension stress is complex and the thin film is ductile, a more robust configuration has to be developed. 

**Figure 22:**
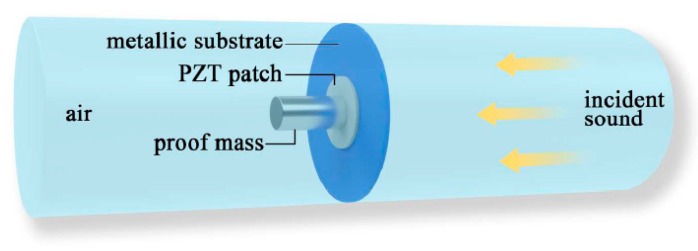
Schematic diagram of the proposed metamaterial structure for AEH [64].

Hence, Yuan et al. [64] utilized a metallic substrate and a proof mass to form a local resonant acoustic metamaterial; this approach did not require tension. A piezoelectric patch was placed inside the proof mass and metallic substrate, converting the structural strain energy into electrical energy. The schematic diagram is shown in Figure 22. A simulation analysis showed that the electromechanical coupling strength was greatly increased after structural optimization. In the experimental study, 200 µW of electrical power was harvested at 155 Hz with 114 dB SPL excitation. Hence, if five units are used simultaneously, harvest power can be up to 1 mW.

**Figure 23:**
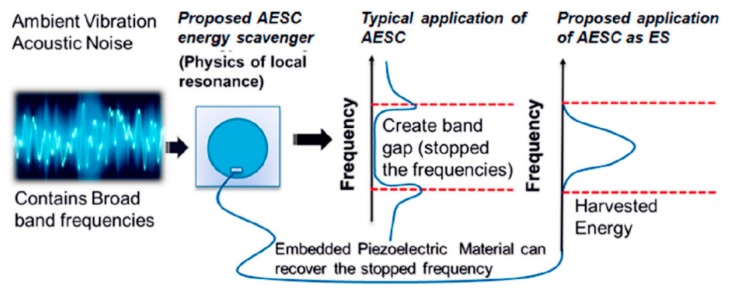
Proposed sonic-crystal structure for noise filtering and energy harvesting. Reproduced with permission from [66]; published by SAGE journals, 2017.

Ahmed and Banerjee [65] and Ahmed et al. [66] proposed sub-wavelength scale acoustoelastic sonic crystals (AESC) to achieve energy harvesting in the low-frequency range. As shown in Figure 23, a PZT wafer is placed inside the unit to convert the strain energy into electrical energy under multiple excitation frequencies. The versatile unit can be adapted to different frequency ranges, and the unit is also capable of simultaneously filtering acoustic waves.

**Figure 24:**
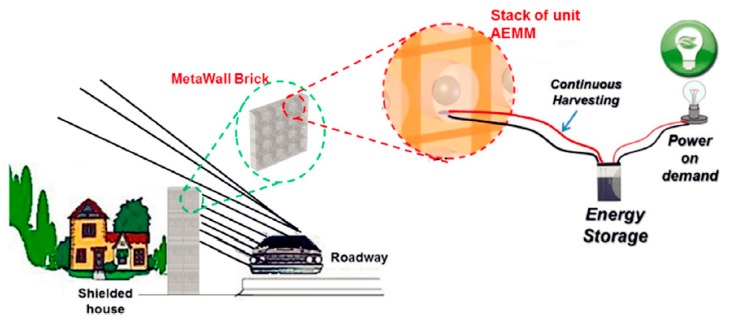
MetaWall structure for noise blocking and energy harvesting. Reproduced with permission from [67]; published by ELSEVIER, 2018.

Later, as shown in Figure 24, multiple AESC units were assembled into an acoustoelastic MetaWall for simultaneous noise blocking and energy harvesting [67]. The simulation results indicated that at 460 Hz, the harvested power was 2 mW, when PVDF material was used. Overall noise-reduction performance was also in good condition.

**Figure 25:**
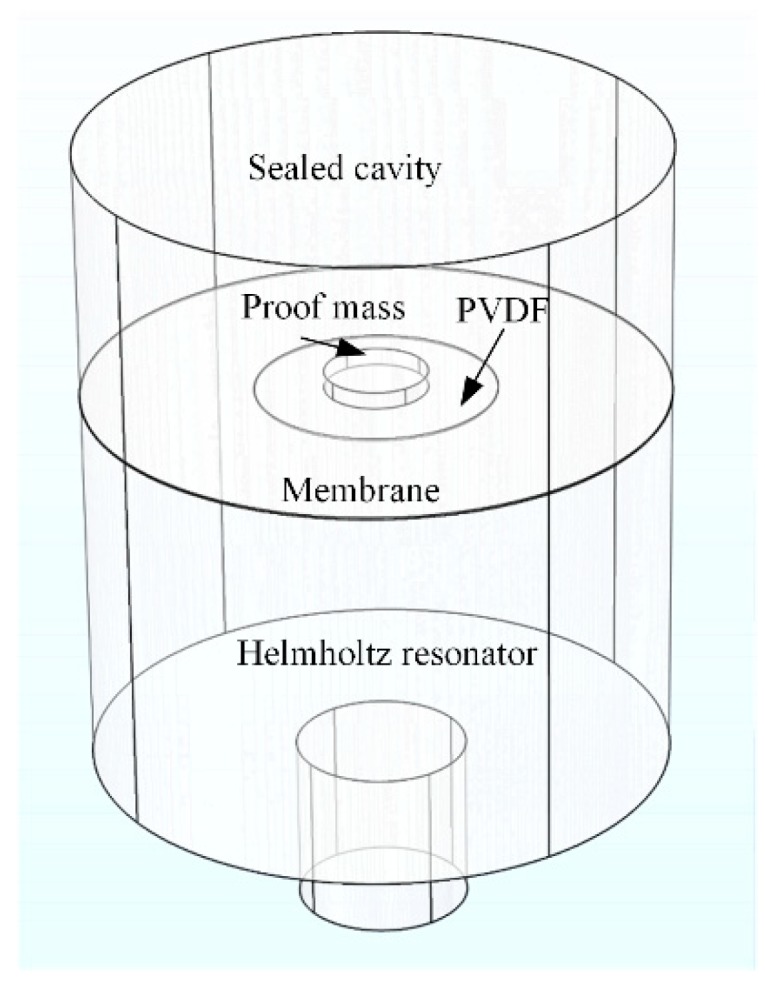
Schematic diagram of the proposed acoustic metamaterial.

Zhang et al. [68] proposed another type of acoustic metamaterial for simultaneous noise insulation and energy harvesting. The proposed structure is composed of a Helmholtz resonator and a built-in decorated membrane; the schematic diagram is shown in Figure 25. The added harvested power was 3.22 µW at 353 and 460 Hz, and two noise-insulation bands were created.

**Figure 26:**
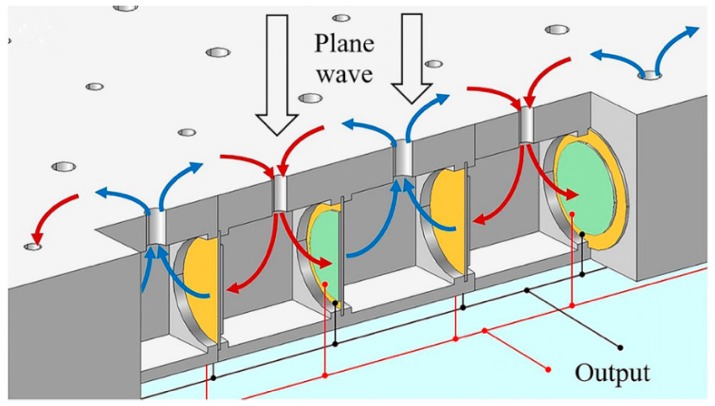
Schematic diagram of the AEH metasurface Reproduced with permission from [69]; published by American Institute of Physics, 2018.

Liu et al. [69] proposed a broadband AEH metasurface, that was composed of coupled Helmholtz resonators and a PZT wafer. A push–pull effect was achieved with different neck lengths of the Helmholtz resonator and resulted in good excitation of the PZT patch in a wide range. The maximum harvested power was 90 mW at 620 Hz with 160 dB SPL excitation. The schematic diagram of the AEH metasurface is shown in Figure 26.

Based on the extraordinary acoustic transmission phenomenon, Cui et al. [70] proposed acoustic grating for AEH. The acoustic field received a strong boost at the resonance stage, and a PZT patch was used to convert the strain energy into electrical energy.

### 4.4. Other Approaches

Other AEH approaches that do not fall into the aforementioned three categories also exist. For instance, Zhou et al. proposed a bistable AEH [71] that generated coherence resonance under acoustic excitation. Under this state, maximum power was harvested. The open-loop voltage reached 0.4 V under 105 dB SPL excitation. However, the power lever was not reported.

**Figure 27:**
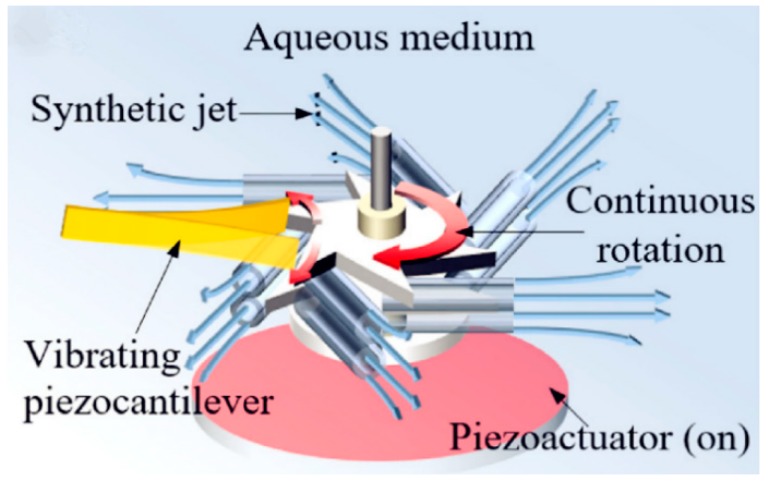
Components of the proposed AEH device. Reproduced with permission from [72]; published by ELSEVIER, 2018.

In another interesting study by Jiang et al. [72], an acoustic bubble that induced a synthetic jet to power the rotor and a piezoelectric beam on the rotor were used for AEH. The schematic diagram of the proposed device is shown in Figure 27.

## 5. Performance Comparison

In this section, the reported AEH performances of the experimental studies are summarized. As shown in Equation (5), the incident acoustic energy is in quadratic proportion to the sound pressure; therefore, a metric is used to compare the performance. This metric is the harvested power normalized by the square of the sound pressure and volume. Because the required information for the metric calculation was not provided in some studies, only the available results are presented.

The detailed AEH performance values are summarized in Table 3. 

In should be noted that although energy performance can be used to judge the overall performance of an AEH device, more focus should be placed on applicable frequency and size.

In general, harvesting low-frequency acoustic energy usually requires a larger device than harvesting mid–high frequency acoustic energy. However, deep subwavelength AEH devices can be designed using acoustic metamaterials, which are more suitable to handle these conditions. In addition, the local resonant property can effectively trap elastic energy, and it can be converted into electric energy via the piezoelectric effect, generating an effective method for energy harvesting.

For instance, although quarter-wavelength tube resonators achieve high power output at a low frequency, tube size may hinder its wide application. An alternative approach is the local-resonant acoustic metamaterial-based AEH [64], which achieves similar performance to the quarter-wavelength tube resonator-based approach but requires less space. 

It has also been shown that resonant coupling is favorable for boosting energy-harvesting performance but it requires a delicate tuning process. In addition, AEH performance markedly differs for piezoelectric materials versus electromagnetic mechanisms, indicating that this topic requires further research. 

## 6. Conclusions

In AEH, acoustic energy is converted into electrical energy, and this method has potential applications for the Internet of Things. An AEH device has low requirements in terms of installation and operation, and can be integrated into other engineering structures to obtain smart structures. 

In this review, the mechanism and potential applications of AEH have been described. The literature survey provides details on the different approaches used in this field that can be categorized into the Helmholtz-based approach, the quarter-wavelength-based approach, and the acoustic metamaterial- based approach. A quantized metric was also provided to evaluate the performance of different AEH devices. 

We have witnessed and participated in the fast development of this exciting technique. In our view, the development of acoustic metamaterials provides fruitful approaches to achieve efficient AEH with compact devices. In addition, the development of novel materials will improve the harvesting performance.

Although many effective prototypes have been developed, a large gap remains between research and actual applications. Multidisciplinary studies and the development of power management and storage techniques are required to close this gap. Moreover, for large-scale deployment, system-level optimization is required, which includes unit-design optimization, circuit optimization for power management and storage, cost optimization, integration with other optimized energy-harvesting techniques, and optimization of the power-consumption device.

## Figures and Tables

**Table 1:** Typical relationships of sound pressure, sound-pressure level (SPL), and sound intensity.

Sound Pressure (Pa)	SPL (dB)	Sound Intensity (W/m^2^)
0.2	80	0.0001
1	94	0.0025
2	100	0.01
10	114	0.25
100	134	25
2000	160	10000

**Table 2:** Properties of commonly used piezoelectric materials. PVDF–Polyvinylidene difluoride, BaTiO_3_–Barium titanate, PZT–Lead zirconate titanate.

Property	Units	PVDF	BaTiO_3_	PZT
Relative dielectric constant	–	12	600–1200	1000–4000
Piezo charge constant $d_{31}$	*pC*/*N*	20	−60–−30	−600–−100
Electromechanical coupling factor	%	11	21	30–75
Young’s modulus	10^10^*N*/m^2^	0.3	11–12	6–9
Density	kg/m^3^	1780	5300–5700	7500–770

**Table 3:** Acoustic Energy Harvesting (AEH) performance comparison.

Incident SPL (dB)	Sound Pressure (Pa)	Volume of Harvester (cm^3^)	Harvested Power (µW)	Metric µW/(Pa^2^·cm^3^)	Frequency (Hz)	Reference
149	563.7	2.445	6 × 10^−^^6^	7.7228 × 10^−12^	13570	[36]
145.5	376	55.5	0.094	1.198 × 10^−8^	834	[37]
100	2	13.57	789.65	14.536	319	[38]
130	63.2	21.2	49	0.0005787	2100	[39]
100	2	735	7.5	0.0026	1324	[43]
100	2	3970	1430	0.09	170	[44]
130	63.2	13.12	214.23	0.0041	1501	[45]
100	2	160	3.49	0.0054	332	[46]
100	2	200	27.2	0.034	217	[47]
113	9	1160	2.2	0.0000234	146	[51]
113	9	840	12700	0.187	199	[52]
100	2	19.44	8.8	0.1132	2257.5	[56]
110	6.3	3027.6	429	0.0036	5545	[55]
100	2	251	7.3	0.0072	183	[60]
100	2	676	0.345	0.0001276	600	[59]
114	10	11.14	210	0.1885	155	[64]
94	1	154.96	3.22	0.021	~360,450	[68]
